# Chemogenetic stimulation of the hypoglossal neurons improves upper airway patency

**DOI:** 10.1038/srep44392

**Published:** 2017-03-10

**Authors:** Thomaz Fleury Curado, Kenneth Fishbein, Huy Pho, Michael Brennick, Olga Dergacheva, Luiz U. Sennes, Luu V. Pham, Ellen E. Ladenheim, Richard Spencer, David Mendelowitz, Alan R. Schwartz, Vsevolod Y. Polotsky

**Affiliations:** 1 Division of Pulmonary and Critical Care Medicine, Department of Medicine, The John Hopkins University School of Medicine, Baltimore, MD, USA; 2 Department of Otolaryngology, the University of São Paulo, São Paulo, Brazil; 3 National Institutes of Health, National Institute of Aging, Baltimore, MD, USA; 4 Department of Pharmacology and Physiology, The George Washington University, Washington, DC USA; 5 Department of Psychiatry, The John Hopkins University School of Medicine, Baltimore, MD, USA.

## Abstract

Obstructive sleep apnea (OSA) is characterized by recurrent upper airway obstruction during sleep. OSA leads to high cardiovascular morbidity and mortality. The pathogenesis of OSA has been linked to a defect in neuromuscular control of the pharynx. There is no effective pharmacotherapy for OSA. The objective of this study was to determine whether upper airway patency can be improved using chemogenetic approach by deploying designer receptors exclusively activated by designer drug (DREADD) in the hypoglossal motorneurons. DREADD (rAAV5-hSyn-hM3(Gq)-mCherry) and control virus (rAAV5-hSyn-EGFP) were stereotactically administered to the hypoglossal nucleus of C57BL/6J mice. In 6–8 weeks genioglossus EMG and dynamic MRI of the upper airway were performed before and after administration of the DREADD ligand clozapine-N-oxide (CNO) or vehicle (saline). In DREADD-treated mice, CNO activated the genioglossus muscle and markedly dilated the pharynx, whereas saline had no effect. Control virus treated mice showed no effect of CNO. Our results suggest that chemogenetic approach can be considered as a treatment option for OSA and other motorneuron disorders.

Obstructive sleep apnea (OSA) is a common disorder affecting 25–30% of the adult population in the Western world^1^ with the prevalence exceeding 50% in obese individuals^2^. It is caused by a loss of lingual motor tone, leading to recurrent upper airway obstruction during sleep, intermittent hypoxia and sleep fragmentation^3^ and substantial cardiovascular morbidity and mortality^4^. Nasal continuous positive airway pressure can relieve upper airway obstruction, although poor adherence limits its therapeutic effectiveness^5^. Implantable hypoglossal nerve stimulators have been developed to maintain pharyngeal patency during sleep^6^ by activating lingual muscles including the genioglossus (GG), a major pharyngeal dilator^7^. This device, however, had a therapeutic effect only in a subset OSA patients^6^. Similarly, pharmacological approaches had limited success^8^.

Recent developments in chemo- and optogenetics suggest novel approaches for treating OSA. Optogenetics entails the expression of light sensitive proteins (i.e., channel rhodopsin-2 (ChR2)) in neurons^9^. Light-activated contraction of a variety of muscles has been demonstrated when ChR2 is deployed in the motor cortex, peripheral motorneurons or skeletal muscles^10^. However, this approach requires illumination of upper airway motorneurons and/or muscles, which is not practical for clinical application. An alternative approach is to deploy *designer receptors exclusively activated by designer drug* (DREADD) in motorneurons with subsequent activation by a unique ligand, clozapine-N-oxide (CNO)^11^. In this study we examined whether such chemogenetic stimulation of hypoglossal motorneurons can increase GG muscle tone and pharyngeal patency.

## Results

**Figure 1:**
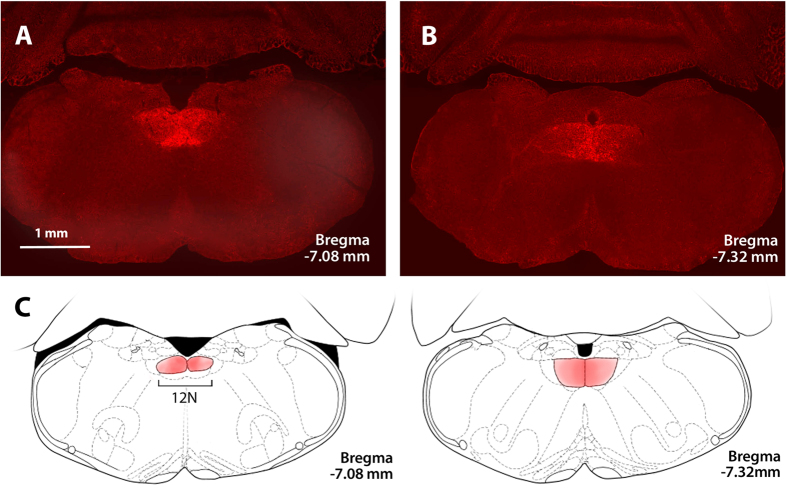
Localization of AAV5-hSyn-hM3 (Gq)-mCherry DREADD in the hypoglossal nucleus. Fluorescent microscopy images (x 10) show mCherry expression spanning the hypoglossal nucleus (**A**) to the internal obex (**B**). (**C**) Localization of DREADDs according to a brain atlas^33^. 12N denotes the hypoglossal nucleus. Credit: Publisher shall credit the image as such: Revised from The Mouse Brain in Stereotaxic Coordinates, Paxinos, George. Figs 92 and 94, p122 and 124. © 2011 Elsevier. Used with permission.

**Figure 2:**
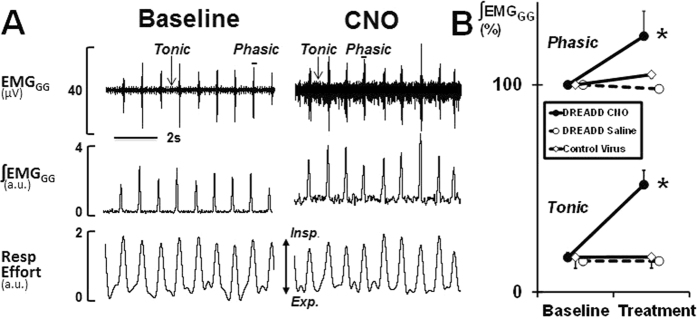
Effect of Clozapine N-Oxide (CNO) on genioglossal muscle activity in mice treated with DREADDs. (**A**) Representative genioglossal electromyography (EMG_GG_), moving average (∫EMG_GG_) and respiratory effort recorded at baseline (left) and after CNO administration (right). Note the robust increase in both phasic and tonic EMG activity after CNO. (**B**) EMG response to CNO or saline in the same DREADD treated animals (n = 13; 15 minutes after injections) and EMG response to CNO in mice treated with control virus (n = 6, 15 minutes after injection) normalized to peak phasic EMG at baseline. a.u. arbitrary units. *, p < 0.001.

Six –eight weeks after infection with rAAV5-hSyn-hM3(Gq)-mCherry, all thirteen mice expressed DREADD throughout the hypoglossal nucleus (Fig. 1). All six rAAV-hSyn-EGFP treated mice showed control virus at the same location (Supplemental Fig. 1). The EMG_GG_ was performed in each DREADD-treated mouse at baseline, after CNO and saline treatments. CNO induced a striking 3.12 fold increase in tonic GG activity, which was observed within 15 min of CNO administration (Fig. 2) and lasted the entire 6 hr experiment in all mice. CNO also induced a 1.23 fold increase in phasic GG activity, but the response varied between mice. In contrast, saline treatment had no effect in the same animals. The specificity of the CNO effect was tested in six mice infected with the control virus and six additional mice, which were not infected. In these animals CNO had no effect on EMG_GG_ (see Fig. 2 for control virus data; uninfected mice not shown).

**Figure 3:**
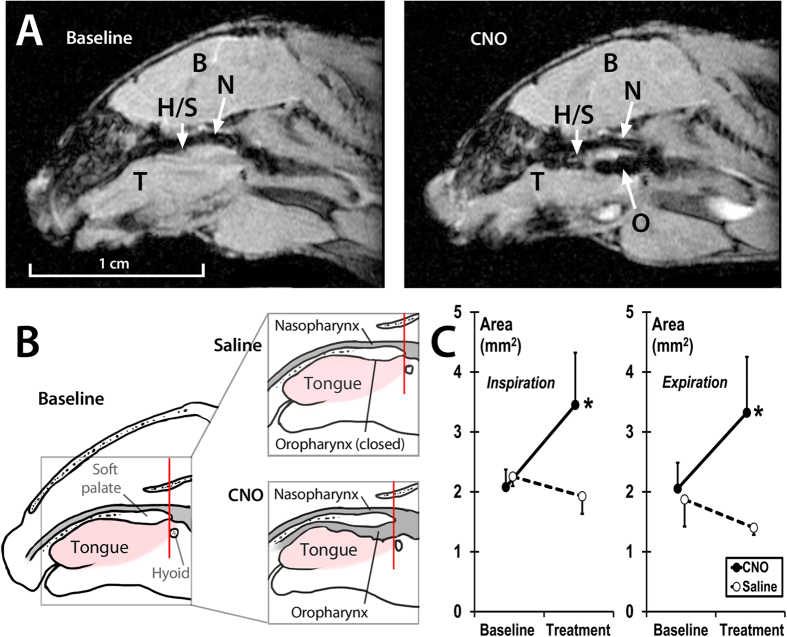
Effect of Clozapine N-Oxide (CNO) on upper airway patency by MRI in mice treated with DREADDs. Representative MR (**A**) and schematic (**B**) sagittal images show dilatation of the upper airway after CNO treatment. (**C**) The cross sectional area of the pharynx on axial images 4 mm caudal to the hard-soft palate junction (red lines on panel B) before and after treatment with CNO (n = 6) show significant dilatation of the pharynx during inspiration and expiration, whereas saline (n = 3) had no effect. *p < 0.05. (**B**) Brain; H/S, the hard/soft palate junction; N, nasopharynx; O, oropharynx. Credit: Publisher shall credit the image as such: Illustration by Corinne Sandone, © 2016 Johns Hopkins University, used with permission.

**Figure 4:**
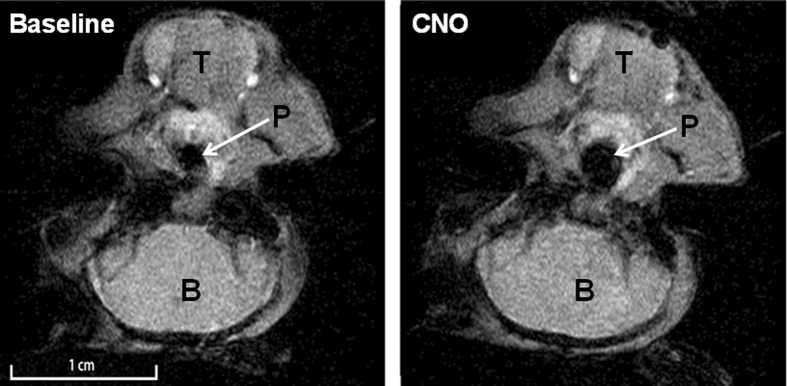
Shows axial MR images of the mouse upper airway 4 mm caudal to the hard-soft palate junction before (left) and after CNO (right). See Fig. 3 for schematic representation of the axial images position (red lines). Note pharyngeal dilatation after CNO treatment. B, brain; T, tongue; P, pharynx.

Nine out of thirteen DREADD-infected mice were examined in a dynamic MR imaging protocol. The pharynx was imaged in the mid-sagittal and multiple axial planes throughout respiratory cycle, both before and after injection of CNO (n = 6) or saline (n = 3) (Fig. 3). Both sagittal and axial dynamic images demonstrated that CNO dilated the pharynx throughout the respiratory cycle. The oropharynx closed at baseline (mice are obligate nasal breathers) was opened by CNO (Fig. 3A,B; Suppl. Fig. 2). At the rim of the soft palate, 4 mm caudal to the hard-soft palate junction, CNO increased the pharyngeal cross-sectional area independent of respiratory phase (p < 0.05), from 2.08 ± 0.29 mm^2^ to 3.45 ± 0.87 mm^2^ during inspiration and from 1.88 ± 0.45 mm^2^ to 3.32 ± 0.93 mm^2^ during expiration (Figs 3C and 4). In contrast, saline injections had no effect on upper airway patency. Three control virus treated mice (n = 3) were examined in the same MRI protocol. CNO had no effect on the upper airway dimensions throughout the respiratory cycle in these animals. For example, the pharyngeal cross-sectional area 4 mm caudal to the hard-soft palate junction during expiration was 2.13 ± 0.99 mm^2^ and 2.09 ± 0.92 mm^2^ before and after CNO, respectively.

## Discussion

To our knowledge, this is the first study examining the effect of chemogenetic stimulation on the upper airway musculature. We report that DREADD-mediated excitation of hypoglossal motorneurons (1) dramatically increased both tonic and peak phasic GG muscle activity and (2) markedly improved upper airway patency throughout the respiratory cycle. Our data may open a new line of research for pharmacotherapy of OSA.

Considerable research effort has been dedicated to the development of pharmacological agents for OSA over the last several decades^8^,^12^. In patients with an anatomic predisposition to OSA due to adiposity or facial anatomy, dilator muscles maintain pharyngeal patency during wakefulness. Sleep leads to a decrease in muscle tone of upper airway dilators, including the GG muscle of the tongue, especially in REM sleep, resulting in OSA^13^. OSA patients also have blunted neuromuscular responses to the upper airway obstruction, which further contribute to nocturnal collapse of the upper airway^14^,^15^. Adrenergic and serotoninergic mechanisms stimulate hypoglossal motorneurons, but serotonin and noradrenaline reuptake inhibitors have not been effective^16^,^17^,^18^. More recently the inward rectifying potassium 2.4 channel (Kir2.4) has been identified as a novel drug target for OSA, but molecules modulating this channel have not been discovered^19^,^20^. The failure of therapeutics to relieve upper airway obstruction prompted us to examine whether artificially engineered receptors can recruit hypoglossal motorneurons.

DREADDs are G-protein coupled human cholinergic receptors which have been chemogenetically engineered to recognize CNO but no naturally occurring mammalian ligands^11^. The excitatory hM3 (Gq) DREADD has been used to modulate neuronal function in multiple studies of feeding behavior^21^,^22^, energy expenditure^23^,^24^, memory^25^, sleep^26^,^27^ and social behavior^28^, but to our knowledge DREADD has not been previously used to activate motorneurons. In the present study, we successfully deployed DREADDs in the hypoglossal nucleus. Specific activation of DREADDs with CNO increased both respiratory related phasic and tonic activity of GG, the main tongue protrudor muscle. EMG_GG_ activation was observed for 6 hours suggesting a lasting effect. An array of protrudors, retractors and intrinsic muscles are involved in the dynamic control of tongue position and pharyngeal patency, and protrudor activation does not always result in the airway opening^29^,^30^,^31^. Despite complex tongue neuromotor control mechanisms, it is particularly significant that CNO produced substantial oropharyngeal dilation, suggesting that chemogenetics can provide a viable approach for treating upper airway obstruction.

Our study had several limitations. First, both EMG_GG_ and MRI were performed in anesthetized rather than sleeping mice. Additional studies will be required to demonstrate that DREADDs can relieve airflow obstruction during natural sleep. Second, we used non-Cre dependent DREADDs, which were expressed non-selectively across cells and motorneuron pools^11^. Upper airway patency could be further improved by stimulating specific lingual muscles^30^ with Cre-dependent DREADDs activated by retrograde viral vectors containing Cre-recombinase^32^. Third, neurophysiological recordings in brain slices to test the CNO effect on the excitability of hypoglossal neurons have not been performed.

Notwithstanding these limitations, our findings imply that DREADDs administered to the hypoglossal nucleus can dilate the upper airway, and suggest that gene therapy with DREADDs may provide novel treatment for OSA. Our findings also suggest potential utility of DREADDs to treat a broad spectrum of motorneuron diseases.

## Methods

### Animals

Twenty five adult male C57BL/6J mice (Jackson Laboratories, Bar Harbor, ME), 8–10 weeks of age and 27.6 ± 0.4 g of weight at the beginning of the experiment were used in the study. Mice were fed with a chow diet and housed in a 22 °C laboratory with a 12-hr light/dark cycle (light phase 9am–9pm). The animals had free access to food and water. The study was approved by the Johns Hopkins University Animal Use and Care Committee and complied with the American Physiological Society Guidelines for Animal Studies. Nineteen mice were used for experiments with viral vectors (n = 13 for DREADD expression and n = 6 for control virus), and six mice were treated with the DREADD ligand clozapine-N-oxide without viral infection.

### Viral vector administration

Mice (n = 19) were anesthetized with isoflurane for induction (2–3% in closed chamber) and placed in the Kopf stereotaxic apparatus (model 963; Kopf Instruments, Tujunga, CA, USA) with mouse adapter VetEquip, and isoflurane vaporizer. Anesthesia was subsequently maintained at 1–2% isoflurane. DREADD (rAAV5-hSyn-hM3(Gq)-mCherry, University of North Carolina Viral Core, 7 × 10^12^ vg per ml, 40 nl; n = 13 mice) or control virus (rAAV5-hSyn-EGFP, University of Iowa, the same concentration and amount; n = 6 mice) were delivered bilaterally using pre-pulled glass micropipettes (Sutter, Novato, California) to the rostral portion of the hypoglossal nucleus of the medulla using the following stereotactic coordinates from the animal’s bregma: 7.20 mm anterior-posterior, 0.20 mm medial-lateral bilateral and 4.75 mm dorso-ventral. The stereotactic coordinates were determined based on The Mouse Brain Atlas^33^.

### Histology

Mice were sacrificed by isoflurane overdose and rapidly perfused with ice-cold 4% paraformaldehyde in phosphate buffered saline (PBS). The brains were carefully removed, postfixed in 4% paraformaldehyde for 24 h at 4 °C and cryoprotected in 20% sucrose in PBS overnight at 4 °C. The next morning, brains were frozen on dry ice and stored in antifreeze solution at −20 °C until further use. The medulla was cut into 30-μm-thick coronal sections on a sliding microtome (Thermo Scientific HM 560; Waltham, MA, USA). The sections were performed via the entire medulla, mounted on glass slides sealed with antifade medium (Vector; Burlingame, CA, USA). Localization of DREADDs in the medulla was confirmed by visualization of mCherry protein expression using Texas Red Filter, whereas localization of the control virus was confirmed by visualization of EGFP expression using FITC filter (Zeiss Axio D.1 microscope Waltham, MA, USA).

### Electromyography of the Genioglossus Muscle (EMG_GG_)

EMG_GG_ recordings were performed 6–8 wks after DREADD or control virus administration. EMG_GG_ was acquired and analyzed as previously described^34^. In brief, mice were anesthetized with isoflurane (2–3%) initially; thereafter, isoflurane was held at 1–2% to maintain a respiratory rate at 1 Hz (0.9–1.1 Hz). The two Teflon-insulated wire hook electrodes (stainless steel, Teflon-coated, full hard, 0.005-in. bare, 0.008-in. coated; A-M Systems, Carlsborg, WA) were inserted in the genioglossus muscle toward the base of the tongue. The bared ends (0.5 mm) were passed through 27G insulin needle and folded at the bevel in hook fashion. The needle directed the intramuscular placement of the hooks and the wires were sutured to the neck musculature to maintain placement.

The EMG_GG_ signal was amplified, band-pass filtered from 30 to 1,000 Hz (alternating-current preamplifier; model P511K, Grass Instruments), and digitized at a sampling rate of 1,000 Hz (LabChart Pro 7). The EMG_GG_ was rectified, and a 100 ms time constant was applied to compute the moving average (LabChart Pro 7). Respiratory effort was monitored with a sensing bladder wrapped around the mouse as previously described^35^.

DREADD infected mice (n = 13) were treated with clozapine-N-oxide (CNO, 1 mg/kg in saline i.p.) and vehicle (saline) four days apart. Control virus infected mice (n = 6) were treated with CNO only. EMG_GG_ was recorded at baseline for at least 1 h before treatment, followed by continuous EMG_GG_ recording for additional 6 hs after intervention. Both treatments were performed only if mice appear healthy and demonstrated normal weight gain, grooming behavior, and normal food and water intake. For quantitative analysis, the tonic (expiratory) and peak phasic (inspiratory) components were measured for 10 randomly selected breaths 15 minutes after CNO or saline injections. Tonic and phasic EMG_GG_ measurements were normalized and expressed as a percent of average peak phasic moving average at baseline.

Another subset of mice (n = 6) was treated with CNO (1 mg/kg in saline i.p.), which was not preceded by viral vector infection (negative control) with EMG_GG_ recorded at baseline and after CNO treatment as described above.

### MR imaging

MRI was performed 6–8 weeks after DREADD (n = 9) or control virus (n = 3) administration. Mice were anesthetized by inhalation of 2.5% isoflurane in oxygen in an induction box, followed by maintenance at 1–2% isoflurane as needed to maintain stable respiration at 1 Hz. A stream of warm air was used to maintain body temperature between 36–38 °C during loading of the mouse into the scanner and while scanning. The mice were loaded into a cradle containing a conical anesthesia mask with the head fixed in place with ear bars. Respiration was monitored using a pneumatic sensor placed between the cradle and one side of the mouse’s abdomen while rectal temperature was measured with a fiber optic sensor (SA Instruments, Stony Brook, NY). The mice were inserted rear feet first and prone into a Bruker Biospec Avance 7 Tesla MRI scanner equipped with a 120 mm actively-shielded gradient/shim coil and 35 mm linear birdcage transmit/receive resonator (Bruker Biospin, Ettlingen, Germany). Guided by initial pilot scans, a single midline sagittal image was acquired to measure the neck angle and to identify the junction between the hard palate and soft palate, which was used as a landmark for defining subsequent axial scans. This image was acquired with a fat-suppressed fast spin echo (RARE) pulse sequence triggered at end-expiration with repetition time TR = 1 breath (769–1000 ms), echo time TE = 8 ms, echo train length ETL = 8, two signal averages, slice thickness 1 mm, field-of-view FOV = 3 × 3 cm (head-foot × anterior-posterior) and matrix size MTX = 128 × 128, resulting in a voxel size of 234 μm × 234 μm. The neck angle was defined as the angle between the ventral margins of the brain and spinal cord. The position of the mice was adjusted outside the magnet, as needed, to achieve a neck angle between 110° and 130°. B0 field map-based shimming was performed to second order over a 1.8 × 2.3 × 5.9 mm voxel centered at the base of the tongue prior to collection of dynamic images. Localized 1H NMR spectroscopy of this voxel was performed with a respiratory-triggered PRESS sequence and yielded a water peak linewidth (full width at half maximum) of 35–45 Hz. Using the same midline sagittal geometry as for the above fast spin echo scan, a dynamic gradient echo scan was performed using a FLASH pulse sequence with parameters TR = 15 ms, TE = 2.5 ms, flip angle FA = 15°, 40 dynamic frames and four signal averages. MR images covered 600 ms of the respiratory cycle, from mid-inspiration to end-expiration. Typical scan time for each single-slice dynamic experiment was 7–10 minutes, depending upon the actual respiration rate. Additional dynamic scans were then performed for an axial slice through the junction of the hard and soft palates and for similar parallel slices centered 1, 2, 3 and 4 mm caudal to this landmark. Dynamic scans of these axial slices were acquired with the same parameters as for the midline sagittal slice, except FOV = 2 × 2 cm (anterior-posterior × left-right) was used, resulting in a voxel size of 156 μm × 156 μm.

After acquiring a complete set of sagittal and axial dynamic scans, the cradle containing the DREADD-infected mice (n = 9) were removed from the magnet, carefully lifted off the cradle and an i.p. injection of CNO (1 mg/kg in saline, n = 6) or saline (n = 3) was performed. The mice were carefully repositioned to ensure a reproducible neck angle, the cradle was reinserted into the magnet and the above imaging protocol was repeated to identify tonic and phasic changes in upper airway geometry in response to CNO. Control virus – infected mice (n = 3) were examined in the same manner, except that only CNO injections were performed. Data were transferred to ImageJ (NIH, Bethesda, MD) for manual delineation and cross-sectional area measurement of the pharyngeal airways in each dynamic frame for each axial slice. All cross-sectional area measurements were performed in a blinded fashion by a single observer (H.P.). Care was taken to match pre-CNO and post-CNO axial slices by identifying landmarks such as the tympanic bullae in each image.

### Analytic Methods

Mixed-effect multivariable linear regression models were developed to examine whether mouse EMG_GG_ amplitude and pharyngeal area in the axial plane significantly changed after treatment while accounting for between-mice variations. Specifically, EMG_GG_ and pharyngeal area averaged over a phase of respiration (inspiration or expiration) were modeled as functions of treatment (CNO vs. saline) and time point (baseline and after treatment). Separate analyses were performed on the tonic and phasic components of the EMG_GG_. Pharyngeal area analysis was stratified by respiratory phase. Analyses were performed with XTMIXED (STATA 12, Statacorp LP, College Station, TX) and R with LME package (www.R-project.org).

## Additional Information

**How to cite this article:** Fleury Curado, T. *et al*. Chemogenetic stimulation of the hypoglossal neurons improves upper airway patency. *Sci. Rep.* **7**, 44392; doi: 10.1038/srep44392 (2017).

**Publisher's note:** Springer Nature remains neutral with regard to jurisdictional claims in published maps and institutional affiliations.

## Supplementary Material

Supplementary Dataset 1

Supplemental Figure 2
