# Supplementary material for: Chemogenetic stimulation of the hypoglossal neurons improves upper airway patency
Source: Sci Rep. 2017 Mar 10;7:44392. doi: 10.1038/srep44392 (PMC5345079; doi:10.1038/srep44392)
Supplement: Supplementary Dataset 1 [file srep44392-s1.doc]

Supplemental Data for the manuscript entitled

‘Chemogenetic stimulation of the hypoglossal neurons improves the upper airway patency’ by

Thomaz Fleury Curado, Kenneth Fishbein, Huy Pho, Michael Brennick, Olga Dergacheva, Luiz U Sennes, Luu V. Pham, Ellen E. Ladenheim, Richard Spencer, David Mendelowitz, Alan R. Schwartz and Vsevolod Y. Polotsky

**Supplemental Figure 1. Localization of AAV5-hSyn-EGFP control virus in the hypoglossal nucleus.**

Fluorescent microscopy images (x 10) show EGFP expression in the hypoglossal nucleus. 12N, hypoglossal nucleus; 4V, fourth ventricle.

**
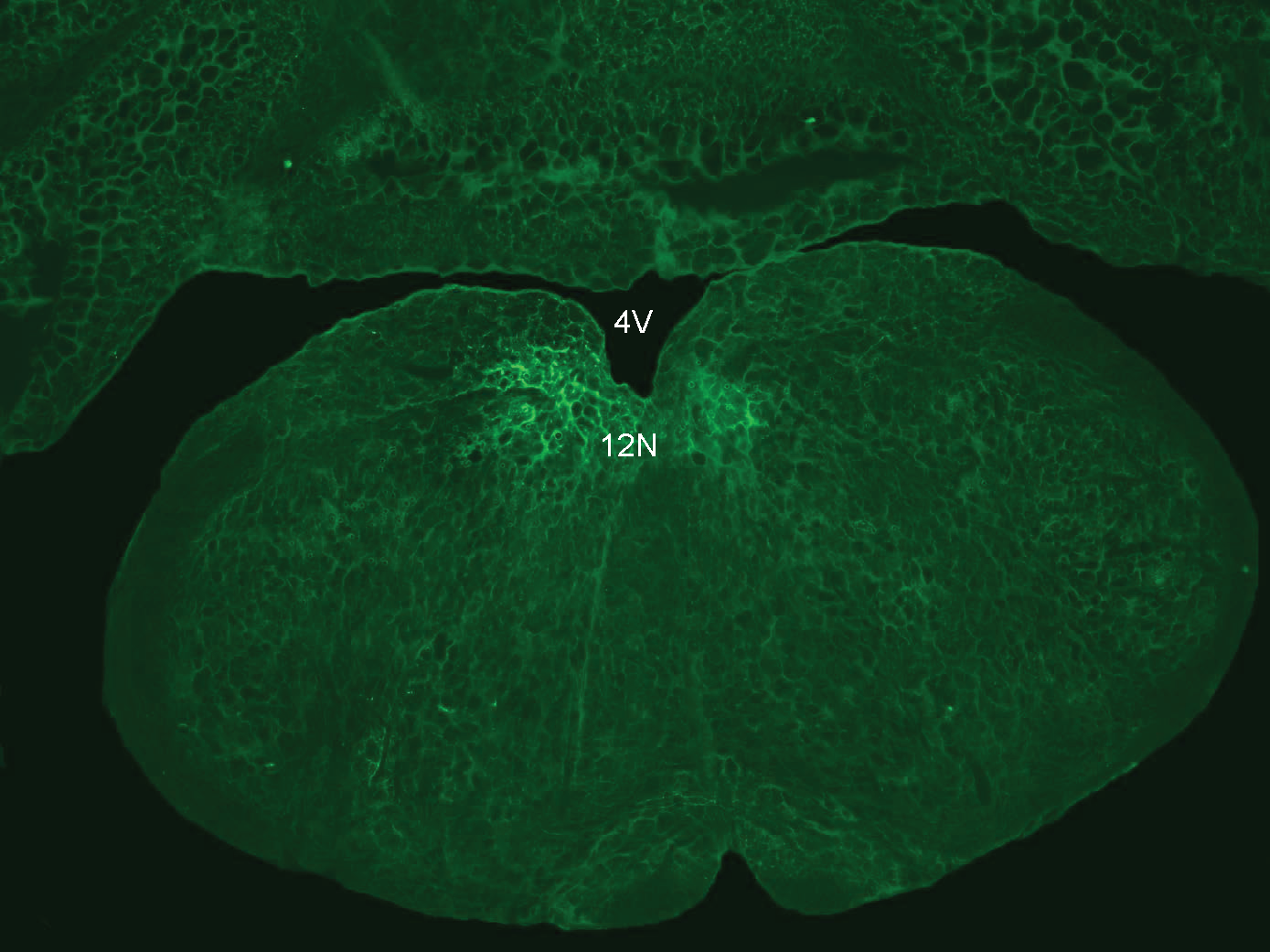
**

**Supplemental Figure 2 (video recording)** shows real time dynamic sagittal MR images of the upper airway of the C57BL/6J mouse 6 weeks after bilateral administration of DREADD (rAAV5-hSyn-hM3(Gq)-mCherry to the hypoglossal nucleus before and after clozapine-N-oxide (CNO) treatment. Note that the oropharynx closed at baseline became widely open after CNO treatment.
